# Electroacupuncture Alters BCI-Based Brain Network in Stroke Patients

**DOI:** 10.1155/2022/8112375

**Published:** 2022-03-10

**Authors:** Zuoting Song, Gege Zhan, Yifang Lin, Tao Fang, Lan Niu, Xueze Zhang, Hongbo Wang, Lihua Zhang, Jie Jia, Xiaoyang Kang

**Affiliations:** ^1^Laboratory for Neural Interface and Brain Computer Interface, Engineering Research Center of AI & Robotics, Ministry of Education, Shanghai Engineering Research Center of AI & Robotics, MOE Frontiers Center for Brain Science, State Key Laboratory of Medical Neurobiology, Institute of AI and Robotics, Academy for Engineering and Technology, FUDAN University, Shanghai 200433, China; ^2^Ji Hua Laboratory, Foshan 528200, Guangdong, China; ^3^Research Center for Intelligent Sensing, Zhejiang Lab, Hangzhou 311100, China; ^4^Yiwu Research Institute of Fudan University, Yiwu 322000, Zhejiang, China; ^5^Department of Rehabilitation Medicine, Huashan Hospital, Fudan University, Shanghai 200040, China

## Abstract

*Goal*. Stroke patients are usually accompanied by motor dysfunction, which greatly affects daily life. Electroacupuncture is a kind of nondrug therapy that can effectively improve motor function. However, the effect of electroacupuncture is hard to be measured immediately in clinic. This paper is aimed to reveal the instant changes in brain activity of three groups of stroke patients before, during, and after the electroacupuncture treatment by the EEG analysis in the alpha band and beta band*. Methods*. Seven different functional connectivity indicators including Pearson correlation coefficient, spectral coherence, mutual information, phase locking value, phase lag index, partial directed coherence, and directed transfer function were used to build the BCI-based brain network in stroke patients. *Results and Conclusion*. The results showed that the brain activity based on the alpha band of EEG decreased after the electroacupuncture treatment, while in the beta band of EEG, the brain activity decreased only in the first two groups. *Significance*. This method could be used to evaluate the effect of electroacupuncture instantly and quantitatively. The study will hopefully provide some neurophysiological evidence of the relationship between changes in brain activity and the effects of electroacupuncture. The study of BCI-based brain network changes in the alpha and beta bands before, during, and after electroacupuncture in stroke patients of different periods is helpful in adjusting and selecting the electroacupuncture regimens for different patients. The trial was registered on the Chinese clinical trial registry (ChiCTR2000036959).

## 1. Introduction

Cerebrovascular diseases are the leading causes of death in the world. Stroke is a kind of cerebrovascular disease characterized by local neurological deficits caused by blood circulation disorders in the brain. Upper limb motor dysfunction is a major problem in the rehabilitation of stroke patients [1]. Upper limb dysfunction can result in limited daily activities in all aspects of having meals, clothing, living, and transportation [2]. It is well known that stroke is a major cause of paralysis [3–5]. Therefore, the rehabilitation of motor function of stroke patients is a research area of great concern at present [2]. There are a lot of methods to assist stroke patients in motor function rehabilitation, including passive and active approaches, while brain-computer interface (BCI) plays an important role in the application of active rehabilitation [6].

Acupuncture treatment is a traditional Chinese medicine approach that is used to help stroke patients recover motor function with the nondrug method [7–9]. It is also a kind of useful treatment to help the other diseases rehabilitation or neurological diseases rehabilitation [10–13]. It is reported that the acupoints such as Quchi, Hegu, and Shousanli are the common and useful acupoints during acupuncture treatment [14, 15]. In recent years, there have been many pieces of research on electroacupuncture treatment for the rehabilitation of upper limb motor function after stroke. However, most of the researches focus on the treatment efforts of the electroacupuncture treatment, not the effect on brain remodeling. On the other hand, there is no good quantitative method to evaluate the immediate therapeutic effect of electroacupuncture.

Most of the studies are to explore the mechanism of electroacupuncture in the treatment of stroke [16–18] and the impact of electroacupuncture on the life of stroke patients [19–22]. In particular, scales are used to evaluate the efficacy of electroacupuncture therapy. Most of the evaluations using scales require relatively obvious improvement effects to have differences in the evaluation results. The high temporal resolution of EEG makes it suitable to evaluate the effect of electroacupuncture in a short time. There have also been several studies of electroacupuncture for stroke patients based on EEG [23–25]. However, most of the methods used in these EEG-based studies are to observe changes in the rhythm, amplitude, or power of the EEG. These basic features of EEG do not characterize changes in specific brain regions, nor do they characterize changes in correlations between brain regions.

It is proved that the brain activity represented by electroencephalogram (EEG) can explain information related to motor function for both the healthy subjects [26, 27] and the patients [28, 29]. Brain connectivity can be used for the study of brain activity [30–32], which is defined as the connection between the different but related parts of the brain in neuroscience [33, 34]. EEG is a high temporal resolution method that can be used to effectively measure brain connectivity [35, 36].

In 2016, it is reported that the synchronization increases at low frequencies and decreases at high frequencies after the transcutaneous acupoint electrical stimulation [37]. In 2017, it is illustrated that the phase synchronization measured by the coherence will have changes in the alpha and beta bands during the acupuncture treatment [38]. In 2018, Yu et al. analyzed the changes of power spectrum and phase lag index before, during, and after acupuncture to study the regulating effect of acupuncture on brain activity [39]. Most of the previous studies only used single index of functional connectivity to evaluate, which cannot reflect the comprehensive effect of acupuncture treatment.

Therefore, the main purpose of this paper is to explore the instant changes in brain activity by exploring the changes of brain networks in stroke patients before, during, and after electroacupuncture. Both the alpha band and beta band of EEG from stroke patients were analyzed, and seven different functional connectivity indexes were used to measure the functional connectivity of the brain. This method is promising to be used as a quantitative measurement of the immediate effect of electroacupuncture treatment.

## 2. Methods

### 2.1. Participants and EEG Recording

The participants aged 18–80 years old involved in the study were from the Department of Rehabilitation Medicine, Huashan Hospital, and Shanghai Third Rehabilitation Hospital. The included subjects (1) were diagnosed with stroke by Computed Tomography (CT) and Magnetic Resonance Imaging (MRI); (2) have no obvious cognitive impairment; (3) have unilateral upper limb hemiplegia and symptoms of weak muscle tone; (4) have no serious comorbidity of osteoarthrosis; (5) are not allergic to EEG electrodes and conductive paste; (6) did not participate in clinical drug trials. According to the above criteria, a total of 18 subjects were selected. According to their lasting time of flaccid paralysis after stroke, they were divided into three groups: the first group is the short-term flaccid paralysis group, and their duration was less than two months; the second group is the mid-term flaccid paralysis group, whose duration was 2–6 months; the third group is the long-term flaccid paralysis group, and their duration was more than 6 months. There are six subjects in each group. The demographic data of the patients is shown in Table S1 (see supporting documents). All subjects included in the experiment received an electroacupuncture treatment. The experiment was approved by the Medical Ethics Committee of Jing'an District Central Hospital of Shanghai (Ethics reference number: 2020–29). The trial was registered on the Chinese clinical trial registry (ChiCTR2000036959).

During electroacupuncture treatment, the subjects' Shousanli, Hegu, and Quchi acupoints were all inserted vertically with acupuncture needles. After the acupuncture needle was inserted, electrical stimulation was performed at a frequency of 2 Hz. The electroacupuncture treatment process took 20 minutes. The EEG signals were recorded using BrainCap 32-channel EEG electrodes at the sampling rate of 1000 Hz. The electrodes were positioned according to the international 10–20 system. The EEG acquisition in the experiment lasted for a total of 30 minutes, including the 5-minute EEG acquisition before the electroacupuncture treatment, the 20-minute EEG acquisition during the electroacupuncture treatment, and the 5-minute EEG acquisition after the electroacupuncture treatment.

### 2.2. EEG Preprocessing

The preprocessing of the EEG signals in the experiment was carried out through the EEGLAB toolbox of Matlab R2020a. We used bandpass filters to divide the frequency bands: alpha band (8–13 Hz) and beta band (14–30 Hz). After that, the EEG signals were rereferenced according to the computed average reference. The EEG signal has a low amplitude and is relatively weak and susceptible to interference. Electrooculogram (EOG) artifacts have a significant impact on the subsequent analysis of EEG signals. Therefore, removing EOG artifacts is a very important step. In the experiment, we chose independent component analysis (ICA) for removing artifacts. After the EEG signal is processed by ICA, the artifact components are automatically identified and removed.

### 2.3. EEG Functional Connectivity Measurement

The study of functional connectivity is a very important part of EEG research. The brain is divided into many brain regions, all of which do not work independently. Most of the tasks are done by different brain regions working together. Therefore, it is important to measure the functional connectivity of the brain. Functional connectivity can mainly be used to measure the degree of correlation between two signals. In the experiment, we used seven functional connectivity indicators to measure brain connectivity.

#### 2.3.1. Pearson Correlation Coefficient

The Pearson correlation coefficient is the simplest of all the indicators of brain connectivity. Although the Pearson correlation coefficient can be used to characterize the correlation between two signals, it is generally only used to characterize the linear correlation.

The Pearson correlation coefficient is defined as the ratio of covariance to standard deviation. Let *X*={*X*_1_, *X*_2_,…, *X*_*N*_},  *Y*={*Y*_1_, *Y*_2_,…, *Y*_*N*_} be two EEG signals with N timepoints. The Pearson correlation coefficient *r* of *X* and Y is [40]

(1) $$\begin{array}{c} r=\frac{\mathrm{cov}\left(X,Y\right)}{\sigma_{X}\sigma_{Y}}\\ =\frac{E\left[\left(X-\mu_{X}\right)\left(Y-\mu_{Y}\right)\right]}{\sigma_{X}\sigma_{Y}}\\ =\frac{\sum_{i=1}^{N}\left(X_{i}-\bar{X}\right)\left(Y_{i}-\bar{Y}\right)}{\sqrt{\sum_{i=1}^{N}{\left(X_{i}-\bar{X}\right)}^{2}}\sqrt{\sum_{i=1}^{N}{\left(Y_{i}-\bar{Y}\right)}^{2}}}, \end{array}$$

where $\bar{X}$ and $\bar{Y}$ are the mean values of *X* and *Y*, and *σ*_*X*_ and *σ*_*Y*_ are the standard deviation of *X* and *Y*, separately.

The Pearson correlation coefficient *r* is in the range of [−1, 1]. The Pearson correlation coefficient can be used to measure whether two signals are positively or negatively correlated. The greater the absolute value of the Pearson correlation coefficient, the higher the correlation between the two signals.

#### 2.3.2. Spectral Coherence

Spectral coherence is also a common index to measure the correlation between two signals. Spectral coherence measures the degree to which two signals are related in the frequency domain. Similar to the Pearson correlation coefficient, spectral coherence can only be used to evaluate the degree of linear correlation between two signals.

The spectral coherence Coh of *X* and *Y* at the frequency *f* where *X* and *Y* are two EEG signals is defined as [41]

(2) $$\begin{array}{c} Coh=\frac{{\left|{Ρ}_{XY}\left(f\right)\right|}^{2}}{{Ρ}_{XX}\left(f\right)\cdot {Ρ}_{YY}\left(f\right)}, \end{array}$$

where Ρ_*XY*_ is the cross-spectral density of *X* and *Y*, and *P*_*XX*_ and *P*_*YY*_ are the auto-spectral densities of *X* and *Y*, respectively.

The values of the spectral coherence are in the range of [0, 1]. The increase of spectral coherence value represents the increase of the correlation degree of signal *X* and *Y* at frequency *f*. When the signals *X* and *Y* are completely unrelated, the spectral frequency coherence index is 0.

#### 2.3.3. Mutual Information

Mutual information is an effective information measurement method based on information theory. Mutual information is a measure of the degree of interdependence among random variables. Different from the Pearson correlation coefficient and spectral coherence index introduced above, the mutual information index can measure not only the linear correlation between two signals, but also their nonlinear correlation. This is the most prominent advantage of it relative to the above two indicators.

The mutual information of two random variables is defined as the relative entropy of their joint and independent distributions. Assuming that *X* and *Y* are two random variables, then their mutual information index *I*(*X*; *Y*) is [42, 43]

(3) $$\begin{array}{c} I\left(X;Y\right)=H\left(X\right)-H\left(X|Y\right)\\ =H\left(X\right)+H\left(Y\right)-H\left(X,Y\right)\\ =\sum_{x,y}P_{XY}\left(x,y\right)\log_{2}\frac{P_{XY}\left(x,y\right)}{P_{X}\left(x\right)P_{Y}\left(y\right)}, \end{array}$$

where *H*(*X*) is the entropy of the variable *X*, *H*(*X|Y*) is the relative entropy of *X* and *Y*, *P*_XY_ is the joint probability of *X* and *Y*, *P*_*X*_ is the probability of *X*, and *P*_*Y*_ is the probability of *Y*.

The mutual information calculated by the above formula is real numbers greater than or equal to 0. In practical applications, it is also a very important step to normalize mutual information. The normalized mutual information NMI(*X*; *Y*) is

(4) $$\begin{array}{c} \text{NMI}\left(X;Y\right)=\frac{2I\left(X;Y\right)}{H\left(X\right)+H\left(Y\right)}. \end{array}$$

The normalized mutual information is in the range of [0, 1]. Although mutual information has certain advantages over the above two indicators, the value of mutual information is easily affected by noise and signal length.

#### 2.3.4. Phase Locking Value

Different from the above indicators, the phase locking value is a phase-based indicator for evaluating the functional connection of the brain. The phase locking value is mainly used to measure the phase difference of the signals and characterize the synchronization of the signals [44]. The phase locking value (PLV) of *X* and *Y* at time *t* where *X* and *Y* are two EEG signals is defined as [44]

(5) $$\begin{array}{c} {\text{PLV}}_{t}=\frac{1}{N}\left|\sum_{t=1\;}^{N}e^{i\left(\phi_{xt}-\phi_{yt}\right)}\right|, \end{array}$$

where *N* is the number of timepoints, *ϕ*_*xt*_ represents the phase value of the signal *X* at time *t*, and *ϕ*_*yt*_ is the phase value of the signal *Y* at time *t*.

The value range of PLV is [0, 1]. The increase of PLV represents the enhancement of the phase synchronization of the two EEG signals. PLV is a commonly used indicator to measure functional connectivity of the brain, but it has an obvious disadvantage that it is susceptible to volume effects.

#### 2.3.5. Phase Lag Index

Similar to PLV, the phase lag index (PLI) is also a phase-based measure of functional connectivity. PLI is an indicator to measure the asymmetry of phase difference distribution between two signals [45]. PLI can also be used to measure the degree of phase synchronization between two signals. The phase lag index is defined as [45]

(6) $$\begin{array}{c} \text{PLI}=\left|\text{sign}\left[\Delta \phi \left(t\right)\right]\right|\\ =\frac{1}{N}\left|\sum_{n=1}^{N}\text{sign}\left[\Delta \phi \left(t_{n}\right)\right]\right|, \end{array}$$

where *N* represents the number of timepoints, sign() represents signum function, and Δ*ϕ*(*t*_*n*_) is the phase difference between two signals at the time *t*_*n*_.

PLI values range from [0, 1]. The larger the PLI value, the more synchronous the phase between the two EEG signals. Different from PLV, PLI calculations are not susceptible to volume conduction effects [46, 47], but PLI is sensitive to noise.

#### 2.3.6. Partial Directed Coherence

Partial directed coherence (PDC) is considered as a multivariable functional connection measurement method based on Granger causality [48]. None of the functional connectivity indicators introduced above is directional. However, PDC measures the causal relationship between signals, so it is directional.

Let *X*(*t*)={*X*_1_(*t*), *X*_2_(*t*),…,*X*_*N*_(*t*)}^*T*^ be the EEG signal with N channels. It can be represented with a multivariable autoregressive (MVAR) model [48]:

(7) $$\begin{array}{c} X\left(t\right)=\sum_{r=1}^{p}A_{r}X\left(t-r\right)+W\left(t\right), \end{array}$$

where *p* represents the order of the MVAR model, *A*_*r*_ is the MVAR coefficient, and *W*(*t*) is the white Gaussian noise.

By Akaike information criterion, *p* can be calculated. *A*_*r*_ can be obtained with the Yule-Walker equation. *A*_*r*_  is transferred to *A*(*f*) in the frequency domain using the Fourier transform. *A*(*f*) can be defined as [48]

(8) $$\begin{array}{c} A\left(f\right)=I-\sum_{r=1}^{p}A_{r}e^{-2jfr\pi }, \end{array}$$

where *I* is the identity matrix. The PDC from channel *j* to channel *i* at frequency *f* can be calculated as [48]

(9) $$\begin{array}{c} \text{PDC}\left(i,j,f\right)=\frac{A_{ij}\left(f\right)}{\sqrt{\sum_{k}{\left|A_{kj}\left(f\right)\right|}^{2}}}. \end{array}$$

The PDC values are in the range of [0, 1]. The larger the value of PDC, the stronger the information flow from channel *j* to *I*.

#### 2.3.7. Directed Transfer Function

Directed transfer function (DTF) is also a kind of connectivity measurement method based on Granger causality. Just like PDC, DTF is directional. At the same time, DTF can be calculated with the similar method used to calculate PDC. The DTF from channel *j* to channel *i* can be defined as [49]

(10) $$\begin{array}{c} \text{DTF}\left(i,j,f\right)=\frac{H_{ij}\left(f\right)}{\sqrt{\sum_{k}{\left|H_{kj}\left(f\right)\right|}^{2}}}, \end{array}$$

where *H*(*f*) represents the inverse matrix of *A*(*f*). Although DTF is very similar to PDC, PDC only detects the direct connection between channels, whereas DTF may detect the indirect connection between channels. This is the disadvantage of DTF relative to PDC, which may lead to a false connection.

The calculation of the seven kinds of functional connection indicators is implemented with MATLAB R2020a. Among them, the calculation of spectral coherence, PLV, and PLI is carried out by SIFT toolbox.

### 2.4. Graph Theory

After calculating all the functional connection indicators, we present the results in the form of the brain network diagram. The graph of each functional connection metric mainly includes two factors: node strength and edge weight.

The weight of the edge represents the absolute value of the corresponding indicator. At the same time, the edges of MI, spectral coherence, the Pearson correlation coefficient, PLV, and PLI are not directional, while the edges of PDC and DTF are directional. The directionality of the edges of PDC and DTF represents the flow of information between channels.

To some extent, the node strength represents the importance of the node in the brain network. For directional indicators, the strength of a node is the sum of the outgoing degree and incoming degree of the node. For nondirectional indicators, the strength of a node is equal to the sum of the weights of the edges associated with it that exceed a certain threshold. The thresholds of different functional connectivity indicators were selected according to the specific conditions of patients in the three groups. At the same time, the different colors of the nodes also indicate the strength of the nodes. The stronger the node is, the greater the index of the corresponding color in the color bar will be.

## 3. Results

In the experiment, three groups of patients with different duration of flaccid paralysis were treated with electroacupuncture, and seven different kinds of functional connectivity indicators were analyzed before, during, and after electroacupuncture. Connections that exceed the set threshold are considered as the significant connections that we need to focus on.

### 3.1. Pearson Correlation Coefficient

The Pearson coefficient is the simplest indicator to measure the functional connectivity between the signals.  Figure S1 of the supporting document illustrates the functional connections based on the Pearson coefficient in the beta band of all three groups before, during, and after the electroacupuncture treatment. The gray edges in the figure represent all connections. The blue edges indicate the significant connections where the value exceeds the set threshold. The size and color of the nodes represent node strength. The value of the Pearson coefficient in each group was the mean of each group.

It is found that the first and second groups both have more significant connections and stronger nodes than the third group before, during, and after the electroacupuncture treatment in Figure S1. The numbers of the significant connections and the strength of the nodes of the first group have not changed a lot during and after electroacupuncture compared with those during electroacupuncture, just decreasing a little. The second group has fewer significant connections after electroacupuncture than those before the treatment. However, the third group has stronger nodes in the central region and more significant connections during and after the electroacupuncture treatment than those before electroacupuncture.

### 3.2. Spectral Coherence

The brain network diagram of spectral coherence in the alpha band of the three groups is shown in Figure S2 of the supporting document. As shown in Figure S2, in the alpha band of the second group, there is an obvious increase in the number of significant connections during and after electroacupuncture relative to it before electroacupuncture, while, in the third group, there is a certain decrease in the number of significant connections during and after electroacupuncture. At the same time, the number of significant connections has less change in the first group during electroacupuncture compared to those before electroacupuncture. The number of significant connections has decreased after electroacupuncture. Only patients in the second group, those with flaccid paralysis between two and six months, have an increase in the number of significant spectral coherence-based connections during and after the electroacupuncture treatment.

**Figure 1:**
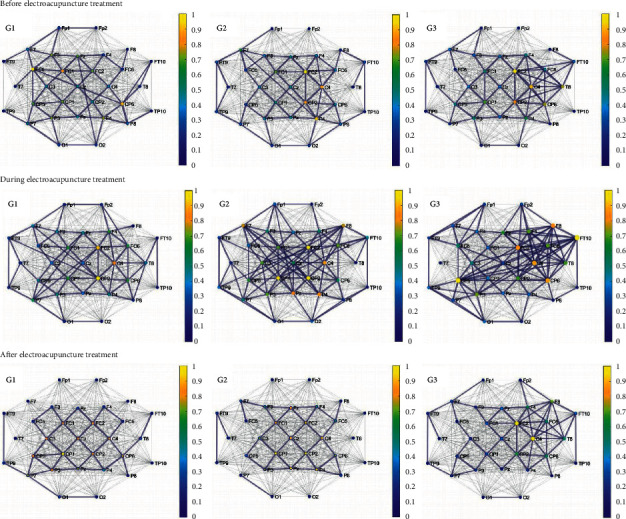
The brain network based on the spectral coherence of the three groups in the beta band before, during, and after the electroacupuncture treatment. G1: the first group, which is the short-term flaccid paralysis group, and their duration was less than two months; G2: the second group, which is the mid-term flaccid paralysis group, whose duration was 2–6 months; G3: the third group, which is the long-term flaccid paralysis group, and their duration was more than 6 months (gray edges: all connections; blue edges: significant connections, the color that corresponds to the colormap and the size of the nodes: node strength).

Figure 1 shows the brain network graph based on spectral coherence in three groups of patients in the beta band before, during, and after electroacupuncture. In contrast to the phenomenon shown in Figure S2, the number of the significant connections during electroacupuncture increased significantly in all three groups compared to the number before electroacupuncture, while the first group had less increase in the number of significant connections, and then they all returned to the smaller number after electroacupuncture than the number before electroacupuncture.

In addition to that, the node color represents the strength of the node. As shown in Figure S2 and Figure 1, the stronger nodes are concentrated in the central region.

### 3.3. Mutual Information

In contrast to spectral coherence, MI can also be used to measure nonlinear connections. As illustrated in Figure S3 of the supporting document, the third group has the fewest number of significant functional connections based on MI in the brain networks before, during, and after electroacupuncture relative to the other two groups in the alpha band.

In the alpha band of the first group, the number of the significant connections during electroacupuncture is distinctly smaller than that before electroacupuncture, and the strength of the nodes was also obviously reduced. However, the number of the significant functional connections and the strength of the nodes both return to a similar state with those before electroacupuncture, only slightly reduced.

As shown in Figure S3, in the second group, the nodes at the left-edged area of the brain network decrease in the strength during electroacupuncture compared to those before electroacupuncture, while most of the nodes at the central and right edged areas increase in the strength. At the same time, the number of significant connections in the left-edged area decreases a little. After electroacupuncture, the node strength and the number of the significant connections in the left and central regions are similar to those before electroacupuncture. However, the strength of the majority of the nodes in the right-edged area obviously increases.

### 3.4. Phase Lag Index

**Figure 2:**
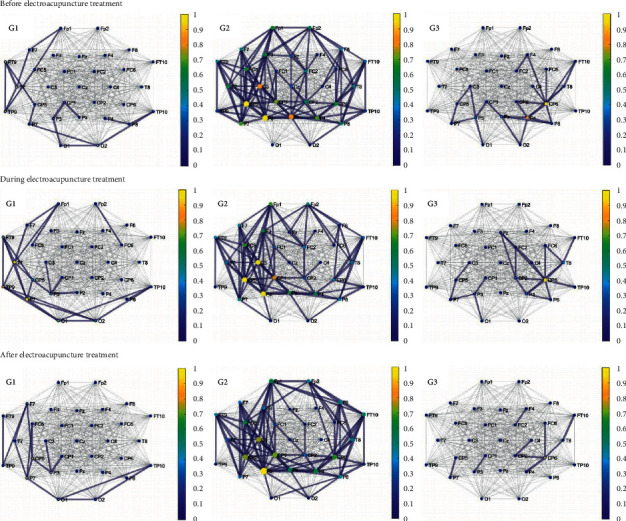
The brain network based on PLI of the three groups in the alpha band before, during, and after the electroacupuncture treatment (gray edges: all connections; blue edges: significant connections, the color that corresponds to the colormap and the size of the nodes: node strength).

It is illustrated in Figure 2 that patients in the second group have the largest number of PLI-based significant connections before, during, and after electroacupuncture in the alpha band relative to those in the first and third groups under the same threshold.

As shown in Figure 2, the number of the significant connections based on PLI and the strength of the nodes in the first group in the alpha band both increase during electroacupuncture compared with those before electroacupuncture. However, the number and node strength both return to the state similar to it before the electroacupuncture treatment.

The number of the significant connections and the strength of the nodes of PLI in the second group decrease during and after the electroacupuncture treatment compared to those before electroacupuncture. Similar results are found in the third group.

### 3.5. Phase Locking Value

The indicator PLV also measures the phase synchronization between the EEG signals.  Figure S4 of the supporting document illustrates the brain network based on the functional connectivity indicator PLV of all the three groups in the beta band. It can be seen in Figure S4 that the first group and the second group have relatively more significant connections than the third group before the electroacupuncture treatment.

During and after the electroacupuncture treatment, the brain network graphs of the three groups all have changed a bit. As shown in Figure S4, the number of significant connections and the strength of the nodes of the first group have not changed a lot, just slightly decreased. According to the three graphs in the second column of Figure S4, it can be seen that the number of the significant PLV connections in the second group has decreased during and after the electroacupuncture treatment.

In contrast to the first and second groups, the number of the significant connections based on PLV and the strength of the nodes of the third group in the beta band both have increased during and after the electroacupuncture treatment compared with those before electroacupuncture. On the other hand, as illustrated in Figure S4, the third group has the most significant connections during the electroacupuncture treatment.

### 3.6. Directed Transfer Function

The most obvious difference between the metrics DTF and those indicators described above is that DTF is directional. Therefore, the brain network map based on DTF is a topological map with directivity.

**Figure 3:**
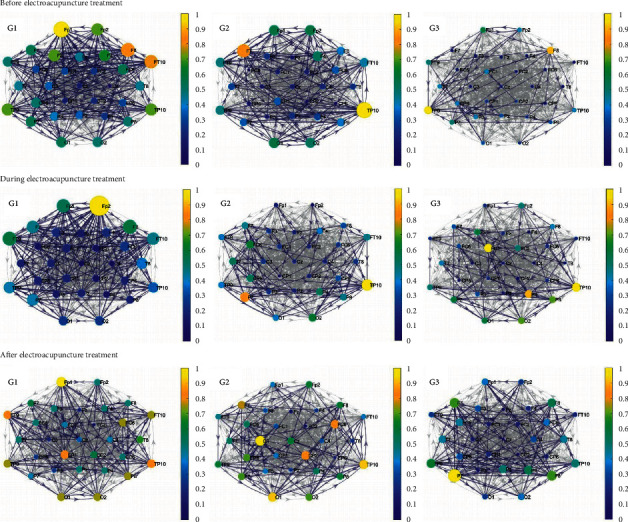
The brain network based on DTF of the three groups in the alpha band before, during, and after the electroacupuncture treatment (gray edges: all connections; blue edges: significant connections, the color that corresponds to the colormap and the size of the nodes: node strength; direction of arrows: information flow).

Figure 3 illustrates the brain network based on the DTF of all the groups in the alpha band. It is can be seen that the first group has the most significant connections and stronger nodes in the alpha band whether before, during, or after the electroacupuncture treatment.

As shown in the first and second columns of Figure 3, the numbers of the significant connections of the first and second groups based on DTF have decreased after the electroacupuncture treatment relative to those before electroacupuncture. Also, the strength of the nodes of the first and second groups has changed a lot during and after the electroacupuncture treatment.

It is also illustrated that the third group has more significant DTF connections and stronger nodes during and after the electroacupuncture treatment than those before electroacupuncture. The number of the significant connections is the largest after electroacupuncture in the third group.

### 3.7. Partial Directed Coherence

PDC and DTF are similar in that they both represent causality and have directivity. Therefore, the brain network maps based on them are all directed topology maps. The general threshold for the PDC indicator is 0.1.

**Figure 4:**
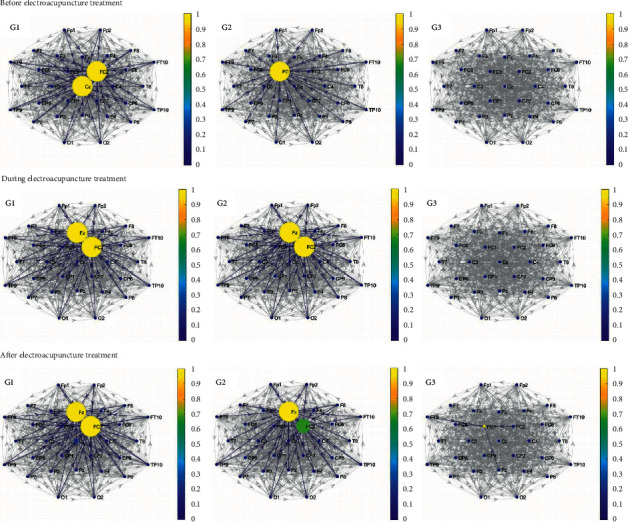
The brain network based on PDC of the three groups in the beta band before, during, and after the electroacupuncture treatment (gray edges: all connections; blue edges: significant connections, the color that corresponds to the colormap and the size of the nodes: node strength; direction of arrows: information flow).

Figure 4 shows the three groups' brain network graphs according to the PDC. It is shown that the number of the significant connections has not changed during the electroacupuncture treatment relative to it before electroacupuncture in the first group. However, the important nodes have changed from Cz and FC2 to Fz and FC2. Among the three groups, the first group has the most significant PDC connections before the electroacupuncture treatment. After electroacupuncture, the number of significant connections increased a little and the important nodes have not changed compared to those during electroacupuncture in the first group.

As illustrated in the second column of Figure 4, the second group has more significant PDC connections and important strong nodes during and after the electroacupuncture treatment than those before electroacupuncture. However, the number of the significant connections and the strength of the nodes both decreased after electroacupuncture compared to those during the electroacupuncture treatment.

It can be seen from Figure 4 that the third group does not have any significant connections based on PDC before and during the electroacupuncture treatment. This situation has changed after electroacupuncture. There is a small number of significant connections after the electroacupuncture treatment in the third group. At the same time, the strength of the nodes has changed a little after electroacupuncture.

## 4. Discussion

Pei et al. used a new wavelet limited penetrable visibility graph (WLPVG) approach to construct the brain network to study the effects of manual acupuncture on the regulation of brain activity in healthy individuals [50]. The power spectral density and simultaneous likelihood of EEG were used to explore the effects of manual acupuncture on the modulation of functional brain activity in healthy individuals [51]. The Pearson correlation coefficient has also been used to study how acupuncture affects the functional network of the brain in healthy subjects [52]. Yu et al. explored the effects of different acupuncture techniques on brain activity in healthy individuals using the brain network based on the phase locking value indicator [53]. The subjects in the studies exploring the effects of acupuncture using indicators of functional brain connectivity were usually healthy subjects, which differed significantly from stroke subjects, and these studies were unable to account for the effects of electroacupuncture on the brain network of stroke patients.

However, most of the studies on electroacupuncture for stroke [23–25] have used methods based on the amplitude and power of the recorded EEG and do not involve the use of functional brain connectivity indicators to assess the brain activity of the subject. In the relevant animal studies, more attention has been paid to the feasibility basis of electroacupuncture for stroke treatment. In contrast, we used the functional connectivity network to more quantitatively assess the effect of acupuncture on brain activity in stroke subjects at different periods and changes in correlation between brain regions. Whereas, in our previous study, we used only partial directed coherence metrics [54], in the paper, we use more comprehensive functional connectivity metrics.

In this research, we experimented with the instant changes of brain network graphs before, during, and after the electroacupuncture treatment based on the functional connectivity of the three groups. In the experiment, seven different functional connectivity indicators including linear correlation index and nonlinear correlation index, directed index, and undirected index were used to construct brain network maps in alpha and beta bands.

It is illustrated that, before, during, and after electroacupuncture, the brain network graphs of all the three groups have a certain change, no matter which indicator was used to map. As shown in Figure S1 of the supporting document, the situation that the number of the significant connections based on the Pearson coefficient in the beta band increased after the electroacupuncture treatment compared to it before electroacupuncture only occurred in the third group whose patients had been paralyzed over six months. As illustrated in Figure S4 and Figure 3, a similar state also appeared in the PLV in the beta band and the DTF in the alpha band.  Figure S1, Figure S4, and Figure 3 show that only the third group had the increase in brain activity after electroacupuncture based on Pearson and PLV in the beta band and based on DTF in the alpha band.

In contrast to the above situation, Figure S2 of the supporting document shows that, for the indicator spectral coherence in the alpha band, only the second group had more significant connections during and after the treatment relative to it before electroacupuncture. It is shown that only the second group had the increase in brain activity based on spectral coherence in the alpha band.

However, it is illustrated in Figure 1 that the numbers of the significant spectral coherence connections of all three groups in the beta band firstly increased during the electroacupuncture treatment and then decreased after the treatment compared to those before electroacupuncture.

Similar to the situation shown in Figure 1, Figure 2 illustrates that all the three groups had fewer significant connections based on PLI in the alpha band after the electroacupuncture treatment compared with those before the treatment. In particular, the first group saw an increase in the number of significant connections during electroacupuncture. It is also shown in Figure 2 that the important nodes of the first and second groups were mostly concentrated in the left parietal lobe and the left central region. It is illustrated that the brain activity based on the spectral coherence in the beta band and based on PLI in the alpha band decreased in all three groups after the electroacupuncture treatment.

Different from the above situation, it is illustrated in Figure S3 that the number of the significant MI connections in the alpha band has not changed after the electroacupuncture treatment compared to it before the treatment in all three groups. In other words, the three groups do not have changes in brain activity based on MI in the alpha band. In contrast to the all above situations, Figure 4 shows that the three groups all had more significant connections and more important nodes based on PDC in the beta band during and after the electroacupuncture treatment than those before the electroacupuncture treatment. The results show that different groups had different changes during and after the electroacupuncture relative to those before the treatment.

The increase in the number of the significant PDC connections in the three groups illustrates the increase in brain activity in the three groups. In addition, the second group showed the greatest increase in brain activity based on PDC in the beta band after electroacupuncture.

It is illustrated that the first group had fewer significant connections based on Pearson, spectral coherence, and PLV after electroacupuncture in the beta band compared to those before the treatment. A similar situation is observed in the second group. However, the numbers of the significant connections based on Pearson, spectral coherence, PLV, and PDC of the third group all increased after the treatment in the beta band. This may indicate that the brain activity in the beta band of the first and second groups decreased after electroacupuncture but increased in the third group. In the alpha band, based on most functional connectivity indicators, brain activity in all three groups showed a downward trend after the electroacupuncture treatment.

The decrease in EEG power in the alpha band was reported in healthy subjects after manual acupuncture [51, 55]. It may characterize the relative sedative effect of electroacupuncture on healthy subjects. Also, it suggests that, in the alpha band, brain activity is reduced in healthy subjects after electroacupuncture. In the paper, it is shown that, based on most of the functional connectivity indicators, patients in whatever period of flaccid paralysis showed a decreasing trend in their brain activity in the alpha band, which is consistent with the experimental effect in healthy subjects. At the same time, our study represents the change more comprehensively and quantitatively and visually in terms of the number of significant connections as well as the degree of the brain network.

It is illustrated that the mean power in the beta band in stroke subjects increased during the needle retention phase and decreased slightly after needle removal compared to before electroacupuncture treatment [25]. However, when studying the effects of electroacupuncture frequency on healthy subjects, the power values in the beta band of the subjects were significantly lower [56]. The result of the paper indicates that brain activity in the third group of patients with a period of flaccid paralysis greater than 6 months rose after electroacupuncture. However, the other two groups showed a decreasing trend. It suggests that changes in beta-band brain activity are influenced by the duration of flaccid paralysis and further suggests that the therapeutic effect of electroacupuncture is also influenced by the duration of flaccid paralysis. The study refines the effects of electroacupuncture more in comparison to previous studies.

The numbers of the significant connections based on the different connections may be related to the threshold value set under each indicator. This may also explain why different indicators based on the same band produce different results. On the other hand, it is suggested that the acupoints, duration, and frequency of acupuncture may have a certain influence on the changes of brain activity before and after acupuncture.

The current disease research is more likely to use classifiers to make predictions about disease. Although, in the paper, brain networks were used to explore the effects of electroacupuncture on stroke patients with different periods of flaccid paralysis, in the future, classification methods such as the Antlion optimization algorithm, implemented on deep neural networks and combined with preprocessing techniques [57], and the fuzzy system based on membership function and fuzzy rules [58] could also be used to investigate the effects of electroacupuncture treatment.

## 5. Conclusion

Electroacupuncture, as a treatment of traditional Chinese medicine, is of great significance in the recovery of motor function of stroke patients. The purpose of this study is to investigate the changes of BCI-based brain networks in the alpha and beta bands in stroke patients at different periods of flaccid paralysis before, during, and after electroacupuncture. There were three groups of patients in the study, those who had been paralyzed for less than two months, two to six months, and more than six months. We constructed brain networks in alpha and beta bands before, during, and after electroacupuncture in three groups of stroke patients based on seven different functional connectivity indicators. The results showed that the brain networks of all groups have changed, regardless of which measure the functional connectivity was based on. For most functional connectivity measures, brain activity in both the alpha and beta bands decreased in the first and second groups. In the third group, brain activity increased in the beta band and decreased in the alpha band. Although our study comprehensively and quantitatively analyzed the effect of electroacupuncture treatment on brain activity in stroke patients with different periods of flaccid paralysis, with only six stroke subjects in each group, the sample size was small, and more subject data are needed to validate the results, and with each subject receiving only one session of electroacupuncture rehabilitation, a single session of electroacupuncture treatment may not produce statistically significant differences in results. Both of these points are limited by the clinical willingness of the patients. We also hope that our study will increase the willingness of patients to receive electroacupuncture treatment. We hope that this work will provide some neurophysiological insight into the relationship between changes in brain activity and the effects of electroacupuncture. The method is promising to be used to quantitatively evaluate the immediate effect of the electroacupuncture treatment. The study on the changes of brain networks before, during, and after electroacupuncture in patients with different flaccid paralysis periods is helpful in further developing and adjusting the electroacupuncture treatment plan for stroke patients.

## Data Availability

The data that support the findings of this study are available upon request from the corresponding author. The data are not publicly available because it would compromise the privacy of the patients involved in the treatment.

## Supplementary Materials

Supplementary Materials Figure S1 and Figure S4 in the supporting documents separately show the changes of brain networks in beta band based on the Pearson correlation coefficient and PLV in three groups of stroke patients before, during, and after electroacupuncture. It is shown in Figure S1 and Figure S4 that the brain activity in the beta band based on Pearson and PLV only increased in the third group. The brain networks based on the spectral coherence and mutual information in the alpha band of the three groups before, during, and after the treatment are described separately in Figure S2 and Figure S3. It is illustrated that brain activity based on spectral coherence in the alpha band only increased in the second group, and there were no obvious changes in brain activity of all the three groups based on MI in the alpha band. The relevant demographics of the three patient groups are shown in Table S1 in the supporting documents.
