# Supplementary material for: Electroacupuncture Alters BCI-Based Brain Network in Stroke Patients
Source: Comput Intell Neurosci. 2022 Mar 10;2022:8112375. doi: 10.1155/2022/8112375 (PMC8930214; doi:10.1155/2022/8112375)
Supplement: Supplementary Materials — Figure S1 and Figure S4 in the supporting documents separately show the changes of brain networks in beta band based on the Pearson correlation coefficient and PLV in three groups of stroke patients before, during, and after electroacupuncture. It is shown in Figure S1 and Figure S4 that the brain activity in the beta band based on Pearson and PLV only increased in the third group. The brain networks based on the spectral coherence and mutual information in the alpha band of the three groups before, during, and after the treatment are described separately in Figure S2 and Figure S3. It is illustrated that brain activity based on spectral coherence in the alpha band only increased in the second group, and there were no obvious changes in brain activity of all the three groups based on MI in the alpha band. The relevant demographics of the three patient groups are shown in Table S1 in the supporting documents. [file 8112375.f1.pdf]

Supporting documents

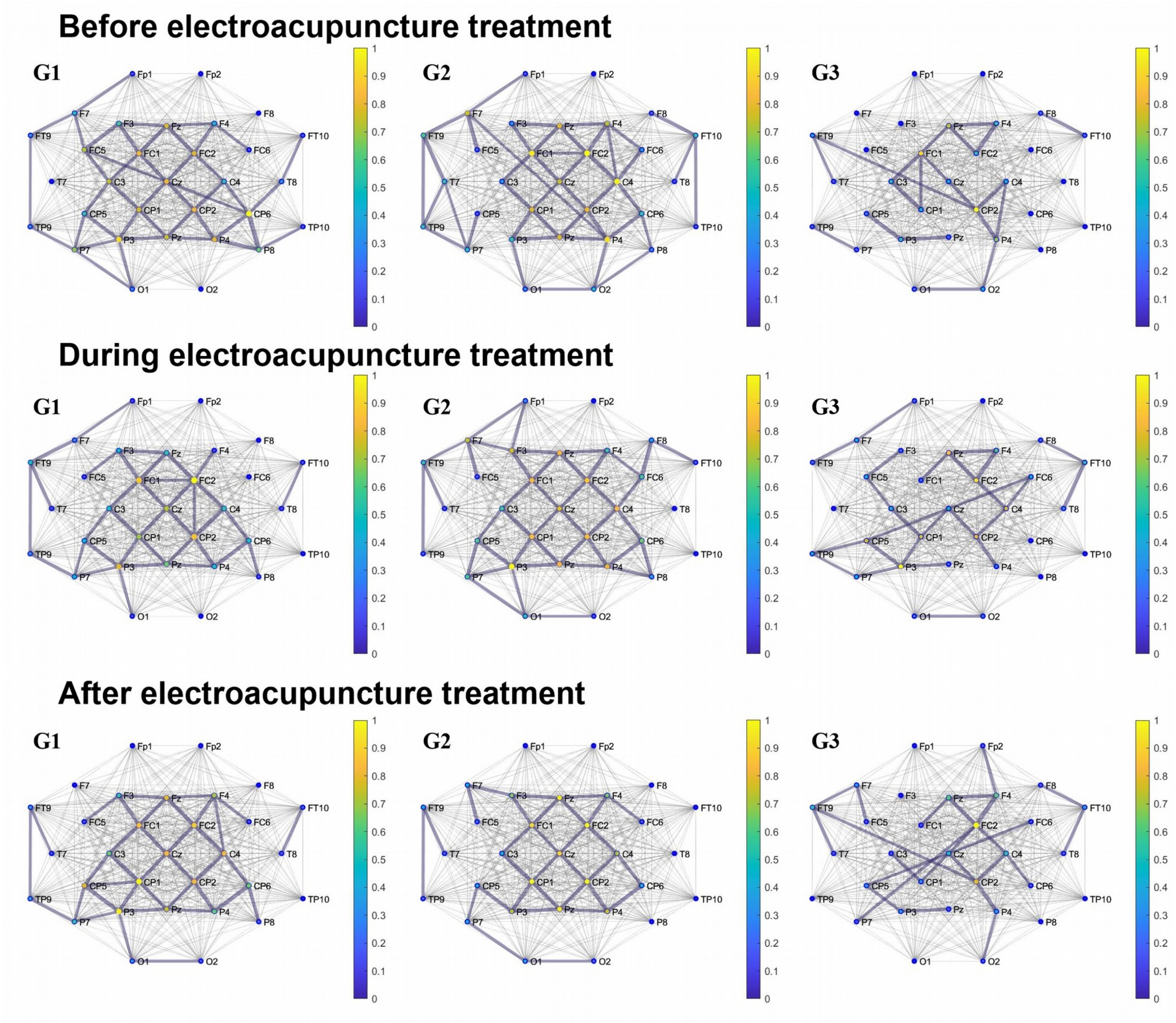

Fig. S1. The brain network based on the Pearson correlation coefficient of the three groups in the beta band before, during and after the electroacupuncture treatment. G1: the first group which is the short-term flaccid paralysis group, and their duration was less than two months; G2: the second group which is the mid-term flaccid paralysis group, whose duration was 2-6 months; G3: the third group which is the long-term flaccid paralysis group, and their duration was more than 6 months. (gray edges: all connections; blue edges: significant connections, the color that corresponds to the colormap and the size of the nodes: node strength)

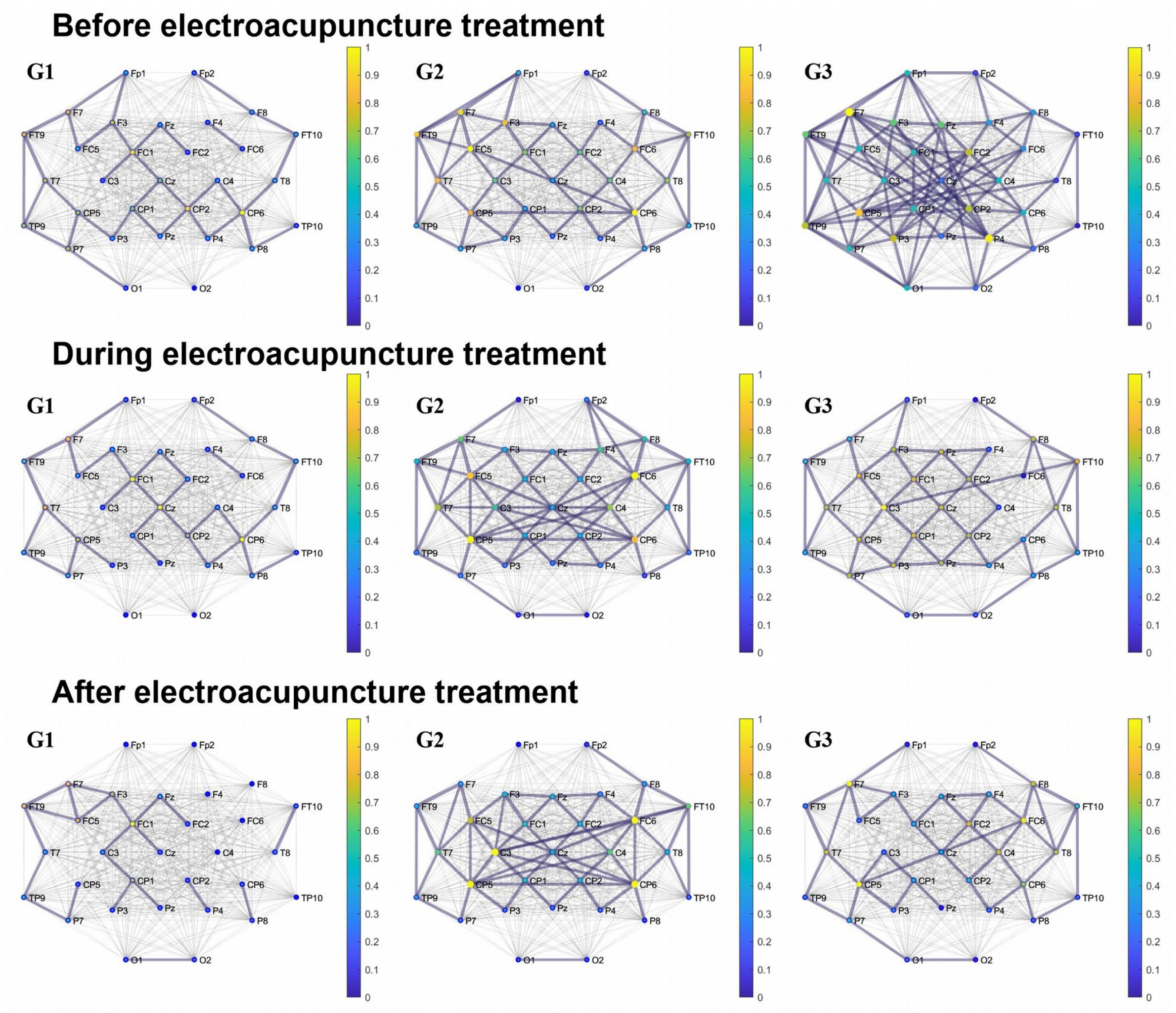

Fig. S2. The brain network based on the spectral coherence of the three groups in the alpha band before, during and after the electroacupuncture treatment. (gray edges: all connections; blue edges: significant connections, the color that corresponds to the colormap and size of the nodes: node strength)

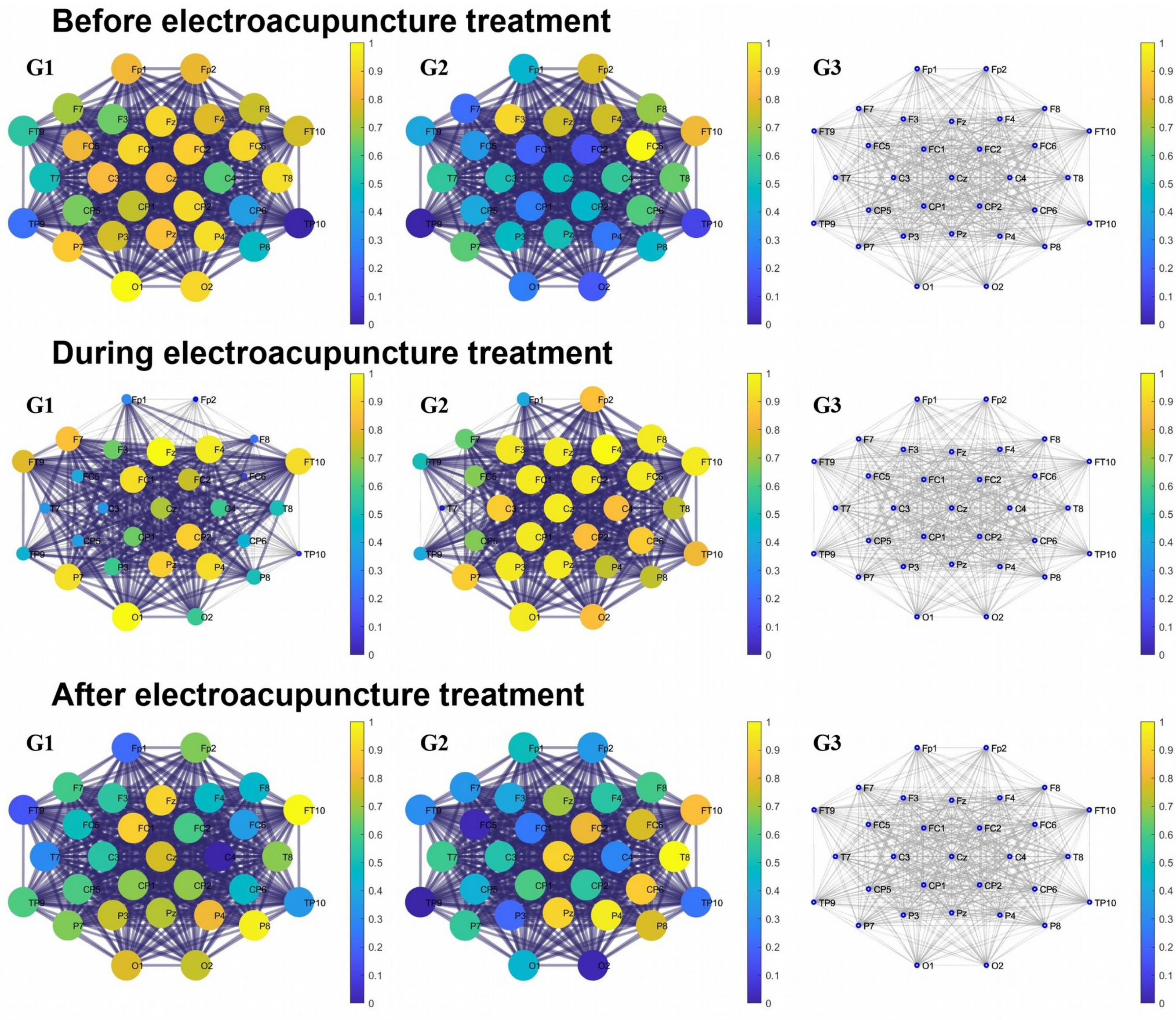

Fig. S3. The brain network based on the mutual information of the three groups in the alpha band before, during and after the electroacupuncture treatment. (gray edges: all connections; blue edges: significant connections, the color that corresponds to the colormap and the size of the nodes: node strength)

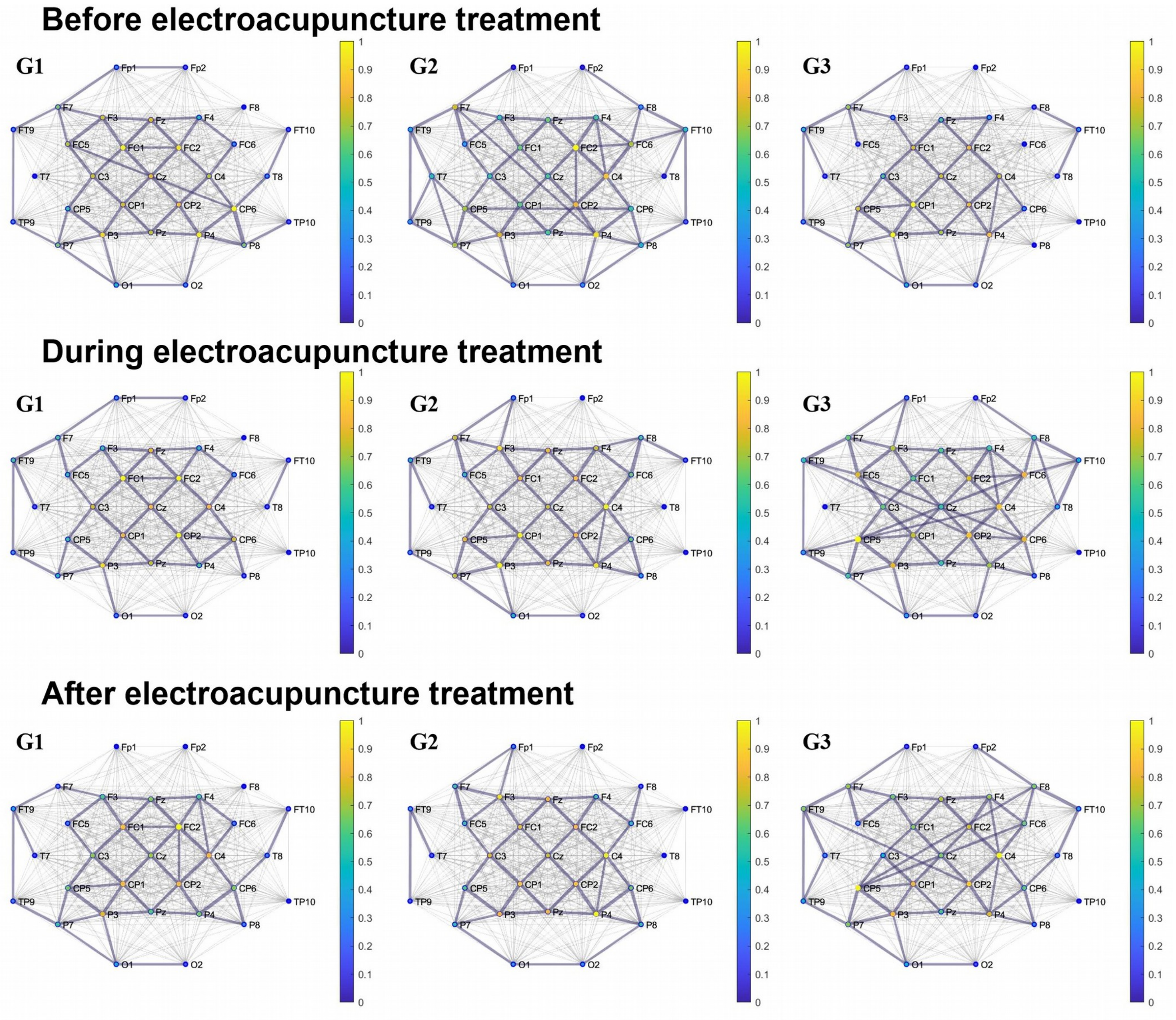

Fig. S4. The brain network based on PLV of the three groups in the beta band before, during and after the electroacupuncture treatment. (gray edges: all connections; blue edges: significant connections, the color that corresponds to the colormap and the size of the nodes: node strength)

Table S1. Demographic data of the patients

Number	Group	Diagnosis	Duration of flaccid paralysis(days)	NIHSS	FMA of Upper limb
01	Short-term group	Ischemia	55	11	4
02	Short-term group	Ischemia	15	11	4
03	Short-term group	Hemorrhage	22	13	6
04	Short-term group	Ischemia	17	12	0
05	Short-term group	Ischemia	11	7	4
06	Short-term group	Ischemia	25	8	4
07	Mid-term group	Ischemia	98	9	5
08	Mid-term group	Hemorrhage	108	4	7
09	Mid-term group	Ischemia	62	14	2
10	Mid-term group	Ischemia	111	9	25
11	Mid-term group	Ischemia	146	8	4
12	Mid-term group	Ischemia	118	15	4
13	Long-term group	Ischemia	938	5	19
14	Long-term group	Ischemia	198	10	6
15	Long-term group	Ischemia	407	10	11
16	Long-term group	Hemorrhage	185	7	10
17	Long-term group	Hemorrhage	1184	29	4
18	Long-term group	Ischemia	370	24	6

*NIHSS: National Institute of Health Stroke Scale, FMA: Fugl-Meyer Assessment
